# Focus on vulnerable populations and promoting equity in health service utilization ––an analysis of visitor characteristics and service utilization of the Chinese community health service

**DOI:** 10.1186/1471-2458-14-503

**Published:** 2014-05-26

**Authors:** Xiaoxin Dong, Ling Liu, Shiyi Cao, Huajie Yang, Fujian Song, Chen Yang, Yanhong Gong, Yunxia Wang, Xiaoxu Yin, Xing Xu, Jun Xie, Yi Sun, Zuxun Lu

**Affiliations:** 1 School of Public Health, Tongji Medical College, Huazhong University of Science and Technology, Wuhan, China; 2 Norwich Medical School, Faculty of Medicine and Health Science, University of East Anglia, Norwich, UK

**Keywords:** Equity in health service utilization, Community health service, Visitor characteristics, Satisfaction

## Abstract

**Background:**

Community health service in China is designed to provide a convenient and affordable primary health service for the city residents, and to promote health equity. Based on data from a large national study of 35 cities across China, we examined the characteristics of the patients and the utilization of community health institutions (CHIs), and assessed the role of community health service in promoting equity in health service utilization for community residents.

**Methods:**

Multistage sampling method was applied to select 35 cities in China. Four CHIs were randomly chosen in every district of the 35 cities. A total of 88,482 visitors to the selected CHIs were investigated by using intercept survey method at the exit of the CHIs in 2008, 2009, 2010, and 2011. Descriptive analyses were used to analyze the main characteristics (gender, age, and income) of the CHI visitors, and the results were compared with that from the National Health Services Survey (NHSS, including CHIs and higher levels of hospitals). We also analyzed the service utilization and the satisfactions of the CHI visitors.

**Results:**

The proportions of the children (2.4%) and the elderly (about 22.7%) were lower in our survey than those in NHSS (9.8% and 38.8% respectively). The proportion of the low-income group (26.4%) was apparently higher than that in NHSS (12.5%). The children group had the lowest satisfaction with the CHIs than other age groups. The satisfaction of the low-income visitors was slightly higher than that of the higher-income visitors. The utilization rate of public health services was low in CHIs.

**Conclusions:**

The CHIs in China appears to fulfill the public health target of uptake by vulnerable populations, and may play an important role in promoting equity in health service utilization. However, services for children and the elderly should be strengthened.

## Background

In 1978, the Declaration of Alma-Ata formally adopted primary health care (PHC) by the World Health Organization (WHO) as the means for providing a comprehensive, universal, equitable, and affordable healthcare service for all countries [1]. Community health service (CHS) is the main form of PHC. Since the 1960s, many countries have attached importance to the organizational construction and the functional expansion of the CHS. For example, CHS facilities in England provide accessible primary care free of charge [2]. Beginning in the 1970s, Canada introduced community-oriented, multidisciplinary CHS, which focused on social justice and equity [3]. South Africa has implemented the policy of “Universal access to PHC for all South Africans” from 1994 and ever since, the community health institutions (CHIs) have contributed to improving the utilization of PHC [4].

In China, patients can usually choose the health service providers they prefer, and pay the chosen provider directly out-of-pocket. The competition stimulates the health service institutions to update facilities and equipment, enlarge scale, enroll excellent physicians by offering liberal salaries and benefits, and adopt a positive attitude towards patients’ preferences [5]. Consequently, superior health resources and patients concentrate at the secondary-tiered and third-tiered health service institutions, while there is a decline in the utilization of PHC institutions. This results in a serious equity problem, especially with regard to low-income patients who cannot afford high costs of health services. Health equity is the absence of systematic and potentially remediable differences in one or more aspects of health across populations or population groups defined socially, economically, demographically, or geographically [6]. Achieving health equity usually involves ensuring that disadvantaged sectors of the population can both afford and gain access to relevant health services. Reducing health inequities is, for the WHO Commission on Social Determinants of Health, an urgent and ethical imperative [7]. In 1997, the Chinese government endeavored to develop a convenient and affordable primary health service -CHS -for the city residents to meet the PHC need of all populations, especially vulnerable populations who experience limited resources and consequent high relative risk of morbidity and premature mortality [8], and to promote health equity.

Chinese CHIs are divided into two levels: health centers and health stations. A health center covers 30,000 to 50,000 residents and can be equipped with inpatient beds, while a station covers about 3,000 residents and isn’t equipped with inpatient beds [5]. Both health centers and health stations provide outpatient service and emergency service. Patients can seek medical services (including diagnosis and treatment, purchasing medicines, and rehabilitation) and public health services (including health check, preventive care, and health education) in CHIs. By the end of 2010, 98% of cities in mainland China have established CHS systems, including 6,903 centers and 25,836 stations in total [9].

For the 21st century, equity in health service utilization remains a major concern, as an important aspect of the health equity. With the rapid development of the Chinese CHS [5,10,11], a more policy relevant question is whether the CHS system in China is doing what it’s designed to do, what’s the effect of CHS system on meeting the PHC need of vulnerable populations, or further what’s the role of CHS system in promoting equity in health service utilization. There is a lack of studies on this issue [12-14]. Thus, we conducted a four-year continuous investigation on the visitors of CHIs with a large sampling size at the national level. By analyzing the visitors’ demographic characteristics, and comparing that with 2008 National Health Services Survey (NHSS) in Chinese cities [15], we aimed to assess whether CHIs attracted vulnerable populations to seek for health services. The NHSS was a sampling survey of visitors of all levels of health institutions nationwide (including CHIs). We also analyzed the service types used by different visitors (especially the vulnerable populations) and their satisfactions. The purposes were to investigate what types of services they were likely to use and whether they were satisfied, and then to grasp what aspects of the services should be improved to better serve the patients.

## Methods

### Questionnaire design and content

The study was approved by the Research Ethics Committee in Huazhong University of Science and Technology, Wuhan, China. Data were collected using a self-administered uniform questionnaire. The questionnaire was developed in three steps. Firstly, a pool of indicators was formed after literature reviews. Secondly, the indicator pool was modified and established through two rounds of Delphi questionnaire requiring quantitative and qualitative answers. Finally, the questionnaire was further revised after a field pretest. The questionnaire included three parts. The first part was demographic characteristics of the visitors. The second part was the utilization of health services. And the third one was whether the visitors were satisfied with certain aspect of health services. The satisfaction was divided into five levels: very satisfying, satisfying, neutral, dissatisfying and very dissatisfying. Only the ones who choose the levels of very satisfying or satisfying were regarded as being satisfied.

### Sampling frame and sampling methods

The sample size of this study was based on the method recommended by the NHSS in China [15], which suggested that a sample of 24 cities and around 20,000 people would be representative of the target population. Cities were classified according to the World Bank classification (Gross National Income (GNI) per capita, Atlas method) [16,17]: the developed eastern cities, less developed western cities and the central cities between the two. The study selected 35 cities, including 14 eastern cities, 9 central cities, and 12 western cities. The cities were further divided into city districts (The district is the administration division under the city, and a city includes 5–7 districts in average). These cities were selected by the multistage sampling according to socio-economic characteristics, city size, and the development level of CHS. Two centers and two stations were randomly chosen in every district of these 35 cities. Thirty visitors of each center and twenty visitors of each station were surveyed by intercept survey method at the exit of CHIs. The same survey was repeatedly conducted in four years: 2008, 2009, 2010, and 2011.

Valid survey data were available for a total of 88,482 CHI visitors in four years, including 20,013 visitors in 2008, 22,711 visitors in 2009, 22,430 visitors in 2010, and 23,328 visitors in 2011. Visitors included in these surveys served as a representative sample of the overall visitors of the Chinese CHS [18,19].

### Data collection and quality control

The study was organized and coordinated by the Chinese Ministry of Health. According to the study protocol, the Health Bureau of each city provided training to senior investigators, and the trained senior investigators provided training to junior investigators. Then the trained junior investigators carried out the survey on the visitors at the exit of CHIs. The junior investigators daily checked the collected questionnaires to correct the logic errors and try their best to replenish the missing items during the survey. According to the rechecking ratio (5%) used in the NHSS [15], the senior investigators randomly selected 5% from the finished questionnaires to check at the end of the survey. The data was double-blindly entered by two different persons.

### Data analysis

A database was set up with EpiData3.0, and data were analyzed by SAS 9.2.

We analyzed the frequencies, compositions, and trends of the main characteristics of CHI visitors in terms of gender, age, and income. The visitors whose age were less than 15, between 15 and 65, and equal to or more than 65 were assigned to group of children, adults and the elderly respectively. Fifty percent of the median or average income is often applied as the low-income line for the country or region internationally [20]. In this study, we applied the 50% of the mean of the family average monthly income to define the low-income group. In order to illustrate whether CHIs attracted vulnerable populations, we compared the visitor characteristics (gender, age and income) in our study with that of the 2008 NHSS in Chinese cities [15]. The NHSS investigated a nationally representative sample of visitors of all levels of health institutions (including CHIs). Chi-square was used to comparatively analyze the association of the service types used and the satisfactions of CHS with main visitor characteristics based on the data of 2011.

## Results

### Main characteristics of CHI visitors

**Figure 1:**
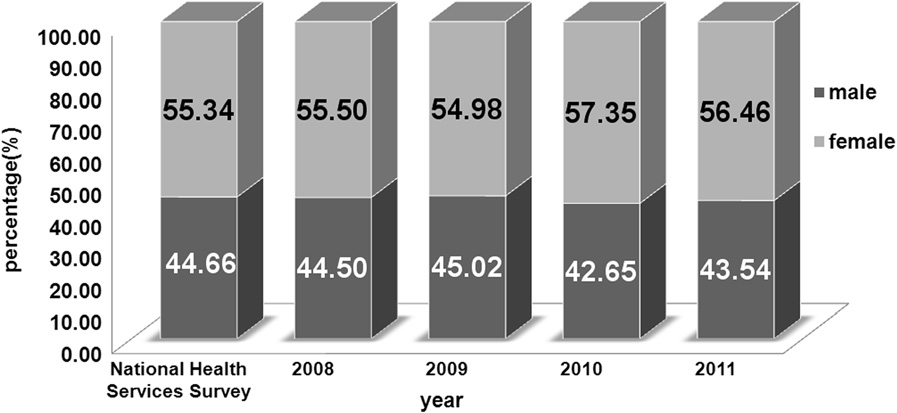
The distribution of the participants by gender.

**Figure 2:**
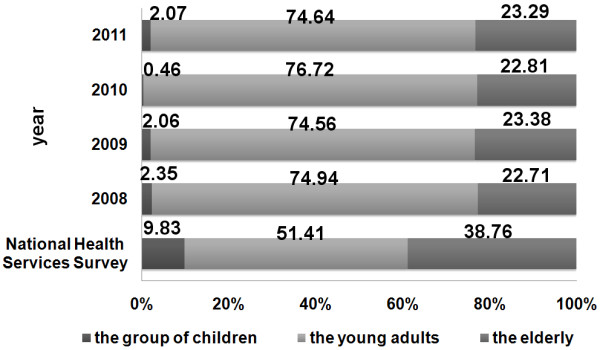
The distribution of the participants by age.

**Figure 3:**
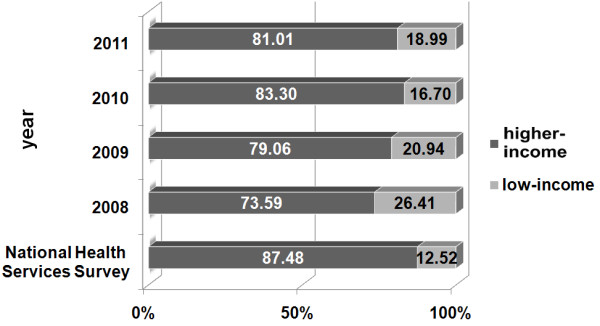
The distribution of the participants by the family average monthly income.

The proportion of male and female visitors was 43.5% and 56.5% respectively in 2011 (Table 1 and Figure 1). The proportion of the children under the age of 15 was 2.4% in 2008, 2.1% in 2009, 0.5% in 2010, and 2.1% in 2011, while the proportion of the elderly visitors over the age of 65 was 22.7%, 23.4%, 22.8%, and 23.3% respectively. The proportions of the children visitors and the elderly visitors were both lower than that estimated by NHSS (Table 1 and Figure 2). The proportion of the low-income patients who used CHIs was 26.4% in 2008, 20.9% in 2009, 16.7% in 2010, and 19.0% in 2010, which was apparently higher than 12.5% in 2008 estimated by NHSS (Table 1 and Figure 3).

**Table 1:** The compositions of the visitors of different characteristics in CHIs and NHSS

		**NHSS in 2008 (city)**		**2008**		**2009**		**2010**		**2011**	
		**n**	**%**	**n**	**%**	**n**	**%**	**n**	**%**	**n**	**%**
**Total**		6474	—	20013	—	22711	—	22430	—	23328	—
**Gender**	**Male**	2892	44.7	8906	44.5	10224	45.0	9566	42.6	10157	43.5
	**Female**	3582	55.3	11107	55.5	12487	55.0	12864	57.4	13171	56.5
**Age**	**0 ~ 14**	636	9.8	471	2.4	468	2.1	103	0.5	483	2.1
	**15 ~ 24**	169	2.6	1356	6.8	1400	6.2	1371	6.1	1355	5.8
	**25 ~ 34**	288	4.5	3304	16.5	3861	17.0	3666	16.3	4193	18.0
	**35 ~ 44**	615	9.5	3528	17.6	3897	17.2	3974	17.7	3844	16.5
	**45 ~ 54**	1013	15.6	3497	17.5	3888	17.1	4082	18.2	3806	16.3
	**55 ~ 64**	1243	19.2	3311	16.6	3889	17.1	4117	18.4	4215	18.1
	**65+**	2509	38.8	4546	22.7	5310	23.4	5117	22.8	5433	23.3
**FAMI**	**Low-income**	811	12.5	5286	26.4	4756	20.9	3745	16.7	4429	19.0
	**Higher-income**	5663	87.5	14727	73.6	17955	79.1	18685	83.3	18899	81.0

CHIs = community health service institutions, NHSS = National Health Services Survey, FAMI = family average monthly income.

The ones whose age were less than 15, between 15 and 65, and equal to or more than 65 were assigned to group of children, adults and the elderly respectively.

### Use of CHI services and visitor characteristics

The use of CHI services was divided into two types: medical services (including disease diagnosis and treatment, purchasing medicines, and rehabilitation) and public health services (including health check, preventive care, and health education).

There were differences in distributions of service types used by different groups of visitors in terms of gender (p < 0.001), age (p < 0.0001), and income (p < 0.05), although the absolute differences were generally small (Table 2). The proportions of male visitors who used medical services were slightly higher than female visitors, while the proportions of male visitors using public health services were slightly lower than female visitors.

**Table 2:** The utilized types of the CHS for different characteristic visitors in 2011

	**Medical services**						**Public health services**					
	**Disease diagnosis and treatment**		**Purchasing medicines**		**Rehabilitation**		**Health check**		**Preventive care**		**Health education**	
	**n**	**%**	**n**	**%**	**n**	**%**	**n**	**%**	**n**	**%**	**n**	**%**
**Gender**												
Male	3954	40.4	2273	23.3	280	2.9	1477	15.1	816	8.4	978	10.0
Female	4998	39.8	2789	22.2	319	2.5	1927	15.4	1261	10.0	1263	10.1
χ^2^, p	χ^2^ = 22.5432, p = 0.0004											
**Age**												
Children	220	49.2	16	3.6	7	1.6	24	5.4	175	39.2	5	1.1
Adults	6576	39.6	3991	24.1	420	2.5	2383	14.4	1604	9.7	1617	9.8
The elderly	2140	40.7	1047	19.9	172	3.3	992	18.9	294	5.6	615	11.7
χ^2^, p	χ^2^ = 766.5750, p < 0.0001											
**FAMI**												
Low-income	1469	40.6	774	21.4	110	3.0	588	16.3	291	8.1	385	10.6
Higher-income	7245	40.2	4085	22.7	466	2.6	2709	15.0	1707	9.5	1797	10.0
χ^2^, p	χ^2^ = 15.5804, p = 0.0081											

CHS = community health service, FAMI = family average monthly income.

The ones whose age were less than 15, between 15 and 65, and equal to or more than 65 were assigned to group of children, adults and the elderly respectively.

The utilization rates of disease diagnosis and treatment services were high for patients of all ages (49.2% for children, 39.6% for adults, and 40.7% for elderly patients). Purchasing medicine services were used mainly by adult patients (3.6% for children, 24.1% for adults and 19.9% for the elderly). The use of rehabilitation services was low for patients of all ages (1.6% to 3.3%). Health check services were mainly used by adult patients (14.4% to 18.9%), and preventive care services mainly used by children (39.2%). Many elderly patients visited CHIs for health education (11.7%).

### Visitors’ satisfaction of CHI services

Female visitors were more satisfied with CHI services than male visitors in terms of their perceptions of privacy protection and avoidance of excessive examination or over-prescription (Table 3). For the different age groups, the elderly group was more satisfied than other groups in convenience, waiting time, environment, service providers’ attitude, respect to patients, equipment, explanation and communication, service and drug prices, and privacy, while the children group was more satisfied in avoidance of excessive examination or over-prescription. As to the different income groups, the low-income group had higher satisfaction in waiting time, environment, service providers’ attitude, respect to patients, equipment, and privacy than the higher-income group.

**Table 3:** The satisfactions of different characteristic visitors with the CHIs (%) in 2011

	**Convenience**	**Waiting time**	**Environment**	**Service attitude**	**Respect to patients**	**Equipment**	**Explanation and communication**	**Service prices**	**Drug prices**	**Privacy protection**	**Avoidance of excessive examination/over-prescription**	**Overall satisfactions**
**Gender**												
Male	98.5	92.0	87.1	94.0	94.0	76.2	92.2	86.5	77.6	87.1	95.0	89.1
Female	98.7	91.7	87.8	94.5	94.4	77.1	92.2	85.9	76.8	88.2	95.8	89.4
χ^2^	1.7557	0.5589	2.4145	3.0453	1.6012	2.5804	0.0009	1.4086	1.8210	6.1338	9.6072	5.1759
p	0.1852	0.4547	0.1202	0.0810	0.2057	0.1082	0.9758	0.2353	0.1772	0.0133	0.0019	0.0229
**Age**												
Children	98.3	83.2	78.6	91.9	92.3	66.8	84.9	69.3	57.7	73.9	96.7	81.2
Adults	98.4	91.2	86.7	93.9	93.7	76.4	91.7	86.1	77.0	87.3	95.3	88.2
The elderly	99.2	94.7	91.0	95.9	95.9	78.7	94.4	88.0	79.6	90.2	96.0	91.2
χ^2^	16.5885	117.7850	105.9991	37.4340	37.8060	39.4893	78.2495	130.4400	121.8400	119.0180	6.6567	495.7900
p	0.0002	<0.0001	<0.0001	<0.0001	<0.0001	<0.0001	<0.0001	<0.0001	<0.0001	<0.0001	0.0359	<0.0001
**FAMI**												
Low-income	98.6	92.7	88.6	94.9	94.5	77.5	92.5	86.0	77.9	88.4	95.5	89.7
Higher-income	98.6	91.1	86.4	93.6	93.9	75.8	91.8	86.4	76.7	87.1	95.9	88.9
χ^2^	0.0426	11.1098	57.4968	11.087	17.1879	5.4979	2.7283	0.3856	2.8197	5.5488	1.8883	5.0385
p	0.8364	0.0009	<0.0001	0.0009	<0.0001	0.0190	0.0986	0.5346	0.0931	0.0185	0.1694	0.0248

CHIs = community health service institutions, FAMI = family average monthly income. The ones whose age were less than 15, between 15 and 65, and equal to or more than 65 were assigned to group of children, adults and the elderly respectively.

In general, the overall satisfactions of female, elderly, and low-income visitors were slightly higher than other corresponding groups (Table 3).

## Discussion

The present study showed that the proportion of female visitors of Chinese CHIs was 55.3% – 57.4%, close to 55.3% reported by NHSS. Similar proportions were reported in other counties such as UK (64.1%) [21], Canada (65.0%) [3], Australia (51.8%) [22], United Arab Emirates (57.0%) [23] and South Africa (66.6%) [24].

The proportion of children in CHI visitors was apparently lower than that of NHSS. A study elsewhere has suggested that the training of doctors for child health care was limited in CHIs [25]. Nowadays, most Chinese families have only one child. Parents attach great importance to their child’s health, and are more likely to seek health care in higher level hospitals when their children get sick [26]. Furthermore, our study indicated that the overall satisfaction of the children group was the lowest. As to the elderly, the proportion was also lower than that of the NHSS. This may be because old patients are more likely to have chronic diseases requiring hospitalization [15], but most CHIs in China do not provide hospitalization service, and then many elderly patients bypass CHIs to go to higher level hospitals for inpatient care service. Moreover, a study elsewhere has found that less than 40% of the chronic patients had been managed in community in China [27], which leads to a large number of chronic patients receiving specialty services in large hospitals rather than in CHIs.

The proportion of CHI visitors with low-income was significantly higher than that reported by NHSS, which indicated that low-income patients tended to use CHS. This may be associated with the payment of service costs. In Canada, patients with lower incomes are less likely to visit specialists than those with moderate or high incomes, after adjusting for self-perceived health status and health problems [28]. In China, the medical insurances mainly cover hospitalization and critical illnesses, the reimbursement rates of which reach 75%, while the reimbursement rate of outpatient costs is only 23% [29]. As a result, the patients still have to pay a larger proportion of outpatient costs. The main objective of Chinese CHIs is to satisfy the basic health service needs of populations, and the outpatient cost in CHIs is substantially lower than that in higher level hospitals. Thus, given the low price, low-income patients are more likely to go to CHIs for outpatient service. In addition, this study showed that the low-income group had higher satisfaction with the CHS. However, both expenditure and service qualities are important. The skill level of the physicians in CHIs is still lower than in higher level hospitals nowadays. Therefore, most patients with high-income may continue to bypass CHIs for perceived higher quality specialist care in high level hospitals [30,31].

The poor, as a vulnerable population according to the international standards, are among the target populations of the Chinese CHS [32]. One of the purposes of developing CHS is to alleviate the problem of low consultation rate of patients, especially vulnerable populations. This study indicated that the low-income patients were more likely to use CHIs for health services, which suggest that developing CHS may attract low-income patients to seek health care, and consequently promote the equity in health service utilization. However, our results also reflect that the Chinese CHIs should strengthen the services for children and the elderly.

The quality of CHS and whether it could meet the demands of patients should be evaluated ultimately by users of the CHIs. The present study found that the visitor satisfactions about CHI services are above 90% in terms of service convenience, providers’ attitude, respect to patients, and avoidance of excessive examinations or over-prescription. However, the level of satisfaction of CHI visitors was only about 75% regarding medical equipment and drug prices. Therefore, equipment of the Chinese CHIs should be much improved in order to meet visitors’ needs. Studies suggested that by way of increasing government investment, improving the compensation mechanisms and medical insurance system, drug prices could be decreased effectively [33,34]. As to avoidance of excessive examinations or over-prescription, care should be taken in the interpretation of the satisfaction values, because the views of health care providers were not taken into account in this investigation.

The overall satisfactions were the lowest among children visitors (81.2%) and the highest in the elderly (91.2%). However, there were no practically meaningful differences in overall satisfaction between females and males, and between low-income and higher-income visitors.

There were small differences in types of health services used in CHIs between visitor groups in terms of gender and income. As to different ages, the utilization rate of preventive care was higher for children, at least partly due to free vaccinations of children in CHIs. Elderly visitors were more likely to use services of purchasing medicine, health check, and health education.

The Chinese government emphasizes the balanced development of basic medical services and public health services provided in CHIs. Nevertheless, the utilization of certain public health services was still insufficient by 2011. The utilization rate of preventive care services was only 9.0%, which was far less than New Zealand (18.9%), Poland (29.1%), America (25.7%), and some other countries [35-37]. This may be attributed to a lack of sufficient appreciation of the importance of the disease prevention in the community [38], or that public health services have not been fully implemented in CHIs. In England, in order to motivate general practitioners to do well in prevention work, general practitioners’ incomes are varied according to the quality and performance of public health services they provide [39]. In Germany, some preventive care services have been covered by medical insurance. In addition, a study suggested that the attitudes towards disease prevention in the community should be changed by health education [40]. China could take similar approaches to improve the utilization of the public health services.

### Limitations

In this study we used intercept survey, which is an economical survey method frequently applied in health service research [41]. Several limitations of our study should also be acknowledged. Firstly, in China, patients could choose health facilities (including CHIs or high level hospitals) for services freely, because of a lack of strict gatekeeper system in community. Findings from this study may be most relevant to China and other countries where there are no gatekeeper systems in community. Secondly, we compared the main characteristics of visitors in our study (represent CHIs) with that of the NHSS, in order to illustrate whether CHIs attracted certain type of patients. The NHSS was a sampling survey of the visitor structure of all level of health institutions nationwide. Thirdly, this was a visitor-based investigation, and the findings could not be related to a population. However, this was a continuous investigation with a large sample size at the national level, which could reflect well the basic situation of CHS in China. Finally, because of the large sample size, some statistically significant differences between groups were small in absolute values.

## Conclusions

A large proportion of low-income group used CHIs for health care services, and CHIs may play an important role in promoting equity in health service utilization. However, the proportions of children and elderly visitors who used CHIs were still low, and services for children and the elderly should be strengthened. Satisfactions of various visitor groups were high in terms of service convenience, providers’ attitude, respect to patients and avoidance of excessive examinations or over-prescription. However, visitors in CHIs had lower satisfactions in terms of medical equipment and drug prices. The children had the lowest overall satisfactions than other age groups. The use of public health services provided by CHIs was still generally insufficient.

## Abbreviations

WHO: The World Health Organization; CHS: Community Health Service; CHIs: Community health institutions; PHC: Primary health care; NHSS: National Health Services Survey; FAMI: Family average monthly income.

## Competing interests

We declare we have no competing interests.

## Authors’ contributions

XD and ZL conceived the idea and prepared a draft review protocol. LL, SC, and HY provided the data and revised the paper. FS provided suggestions for improvements. XD, CY, and YG were involved in the data analysis and write up of the manuscript. YW, XY, JX and YS were responsible for the database and all statistical analysis of data for this paper. All authors read and approved the manuscript.

## Pre-publication history

The pre-publication history for this paper can be accessed here:

http://www.biomedcentral.com/1471-2458/14/503/prepub
